# Mr. Whitlam, on the Management of Leeches

**Published:** 1804-10-01

**Authors:** John Whitlam

**Affiliations:** Nottingham


### *To the Editors of the Medical and Physical Journal.*

Gentlemen,

A Friend of mine having paid considerable attention to the physiology of leeches, and by his method of manageing them has been enabled to preserve them fit for use for several years; has permitted me to communicate the result of his experience, through the medium of your useful and respectable Journal.

It appears that river water, though impregnated with foreign substances, is much preferable to spring water, particularly if it be suffered to stand in a cistern for two or three days previously to being used. It is necessary to give the leeches fresh water every day; and in cold weather, it should be in a tepid state.

The

The vessel containing them must be kept in a place where the temperature will not be reduced below 60° of Fahrenheit.

When you put them into fresh water, you will sometimes observe a greenish excrement come from them, in the form of a thread, which will discolour the water. Whenever this is perceived, it is better to renew the water immediately. You will often see their bodies encircled in various places with pieces of tough, light coloured mucus; this should be removed by gently rubbing them with a soft rag, as they are frequently unable to get rid of it themselves; and, unless this is done, they become sickly, and injure the rest. This seems the most formidable complaint to which they are subject; and on its removal you will be convinced, by the liveliness of their motions, that you have removed an unpleasant and an unhealthy load from them.

If these observations be attended to, and the leeches not too much crouded in the vessel which contains them, there will soon be no reason to complain of their scarcity.

I am, &c.

*Nottingham, Sept.* 6, 1804. JOHN WHITLAM.

